# Silencing of Molt-Regulating Transcription Factor Gene, *CiHR3*, Affects Growth and Development of Sugarcane Stem Borer, *Chilo infuscatellus*

**DOI:** 10.1673/031.012.9101

**Published:** 2012-08-09

**Authors:** Yu-liang Zhang, Shu-zhen Zhang, Mahesh Kulye, Su-ran Wu, Nai-tong Yu, Jian-hua Wang, Hong-mei Zeng, Zhi-xin Liu

**Affiliations:** ^1^ Key Laboratory of Biology and Genetic Resources of Tropical Crops, Ministry of Agriculture, Institute of Tropical Bioscience and Biotechnology, Chinese Academy of Tropical Agricultural Science, Haikou, Hainan 571101, P. R. China; ^2^ Key Laboratory of Integrated Pest Management in Crops, Ministry of Agriculture, Institute of Plant Protection, Chinese Academy of Agricultural Sciences, Beijing 100081, P. R. China

**Keywords:** bacteria-expressed dsRNA, bioassay, ecdysteroid signaling, phylogenetic analysis, quantitative real-time PCR, RNA interference

## Abstract

RNA interference (RNAi) is a technology for conducting functional genomic studies and a potential tool for crop protection against insect pests. Development of reliable methods for production and delivery of double-stranded RNA (dsRNA) is the major challenge for efficient pest control. In this study, *Chilo infuscatellus* Snellen (Crambidae: Lepidoptera) was fed with *CiHR3* dsRNA expressed in bacteria or synthesized *in vitro*. The dsRNA ingested by *C. infuscatellus* successfully triggered silencing of the molt-regulating transcription factor *CiHR3*, an important gene for insect growth and development, and caused significant abnormalities and weight loss in insects within seven days of treatment. This study is an ideal example of feeding-based RNAi mediated by dsRNA expressed in bacteria or synthesized *in vitro*. The results also suggested that feeding-based RNA interference is a potential method for the management of *C. infuscatellus*.

## Introduction

Sugarcane, *Saccharum officinarum* L., is one of the major agricultural crops cultivated in tropical and subtropical regions of the world. The annual production of sugarcane in the world is ∼1.6 billion tons (Krishnan et al. 2010). China is one of the world's largest sugarcane producing countries with the annual production capacity of ∼100 million tons, with an average cane yield of 67 tons/ha. There are many factors which are responsible for low sugarcane yield, among them severe attack of insect pests at early and mature crop stages are the most significant. Sugarcane borers are the most injurious among the pests attacking this crop (Ashraf and Fatima 1993). These include the top borer, *Scirpophaga nivella*; stem borer, *Chilo infuscatellus*; root borer, *Emmalocera depressella*, and Gurdaspur borer, *Acigona steniella*. Borers may reduce the yield up to 80% (Kalra and Sidhu 1955). The damage caused by borers not only reduces crop yield but also affects the sucrose contents of cane. Sugarcane stem borer, *Chilo infuscatellus* (Snellen) (Crambidae: Lepidoptera) is a major pest of sugarcane in China, especially in Guangdong, Guangxi, and Hainan Provinces. The larvae of *C. infuscatellus* feed on the growing point of the sugarcane seedlings, resulting in a withering of seedlings. In usual cases, this single insect species accounts for yield losses of 10–15%, while in severe cases it is reported to cause losses of 40% or more (Arvinth et al. 2010).

The damage is done by larvae which feed inside the cane and are difficult to control with insecticides. Moreover, the heavy use of pesticides creates problems like pest resurgence, outbreak of secondary pests, and environmental pollution. Some special cultural practices and the use of bioagents such as *Sturmiopsis inferens* and *Trichogramma chilonis* have been practiced with limited success (Viswanathan and Samiyappan 2001; Jalali et al. 2006). To overcome these problems, there is an urgent need to find out an alternative strategy to control this insect species.

RNA interference (RNAi) mediated by double-stranded RNA (dsRNA) inducing gene-specific silencing has become one of the most promising methods for studying the function of genes. This technology has also become a potential robust tool for crop protection against insect pests (Gordon and Waterhouse 2007; Price and Gatehouse 2008). Recent studies have described the use of transgenic plants expressing dsRNA to silence the target gene of pests (Baum et al. 2007; Mao et al. 2007; Zhao et al. 2011; Li et al. 2011). In *Caenorhabditis elegans*, an efficient induction of RNAi using bacteria to deliver dsRNA has been developed and successfully used for a rapid and effective genome-wide analysis of gene function (Kamath et al. 2003). This method has used a bacterial strain that lacks the enzyme RNaseIII which degrades dsRNAs in the normal bacterial cell (Fire et al. 1998).

Molting is an important physiological process during the growth and development of an insect. The study of genes related to insect molting helps not only to understand the mechanism of insect development, but also to screen for potential novel biological control strategies. The research on insect molting genes has become the hot topic in insect development biology and pesticide development, which shows the important theoretical and application prospect. The molt-regulating transcription factors HR3 play important roles in regulating expression of tissue-specific genes involved in insect molting. However, most studies on HR3 have been focused on ecdysis regulatory function and molecular mechanism. As the HR3 transcription factors act as regulators in the cascade of molting related genes, it has been selected as a potential target for developing novel approaches for the control of insect pests (Zhao et al. 2004).

In this paper, we first cloned *CiHR3*, a key gene related to growth and development of *C. infuscatellus*, and then used it as an interference target gene The dsRNA was produced using prokaryotic expression system and fed to *C. infuscatellus*. The effect of feeding dsRNA on the growth and development to *C. infuscatellus* was further determined.

## Materials and Methods

### Insect culture

The insect culture of *C. infuscatellus* was obtained from the Sugarcane Farm, Hainan, China. The new field-collected insects were introduced every year in reared insect colonies to maintain genetic diversity at the Institute of Tropical Bioscience and Biotechnoloy, Hainan, China. The insects were reared on sugarcane plants at 25 ± 2 ^°^C and 14:10 L:D photoperiod in insect rearing cages. The number of insects in each cage varied between 10 and 15. Newly planted sugarcane plants were provided twice each week. Egg masses were collected daily and stored in a petri dish with water-soaked filter paper and kept in an incubator (25 ± 2 ^°^C, 70% RH, 16:8 L:D). The young larvae were fed with fresh sweet corn after they emerged in the same petri dishes.

### Cloning of *CiHR3* gene fragment and phylogenetic analysis

The *CiHR3* gene from *C. infuscatellus* was the target gene for RNAi. The total RNA was prepared from the last instar stage of *C. infuscatellus* with Trizol reagent (Invitrogen Life Technologies, www.invitrogen.com). The first strands of cDNAs were synthesized by a PrimeScript™ 1^st^ strand cDNA synthesis kit (Takara, www.takarabio.com). A pair of degenerate primers CiHR3-middle fragment-P1 : 5′- ACA GWG GTG AAC TAC CAG TG -3′, and CiHR3-middle fragment-P2:5′- GAC CAT GRA ATT GGT CGC T -3′ were designed to amplify the homologous nucleic acid sequence by utilizing Primer Premier 5.0 (Premier Biosoft, www.premierbiosoft.com). The primers were based on conserved amino acid regions found in lepidopteran insects. PCR was performed with the following conditions: 94 ^°^C for five min, 35 cycles at 94 ^°^C for 30 sec, 50 ^°^C for 30 sec, and 72 ^°^C for two min, and a final extension at 72 °C for 10 min. The amplified fragment was subcloned into the pMD19-T vector using the TA Cloning kit (Takara, www.takarabio.com), and was subsequently transformed into *Escherichia coli* Top 10 competent cells (TransGen Biotechnologies, www.transgen.com.cn). Nucleotide sequences were determined with an ABI PRISM 3730XL DNA Analyzer using a BigDyeTerminator v3.1 Cycle Sequencing Kit (Applied Biosystems, www.biocompare.com). To clone a longer cDNA sequence, 3′ RACE was performed using gene specific primers for *CiHR3* and adaptor primers supplied with Takara 3′ RACE cDNA Amplification Kit. For 3′ RACE, the gene specific primers that were designed based on the partial amplified fragment mentioned above were: forward specific primer, CiHR3-3′ RACE-GSP1: 5′ -CGG GTC AAC AGG AAC AGG TGT CA -3′, and nested specific primer, CiHR3-3′ RACE-GSP2: 5′- TGT CCA AGA AAC AAC GGG AGA AG -3′. Searching of similar sequences was performed using BlastP in the non-redundant protein sequences database of the NCBI website (www.ncbi.nlm.nih.gov/). It showed homology with 15 related insect molt-regulating transcription factors from different insects. Multiple alignments of the deduced amino acid sequences were conducted by using Clustal X 1.81 software. A phylogenetic tree was constructed by using MEGA4.0.2 software (www.megasoftware.net/) applying the method of Neighbor-Joining (NJ).

### Construction of prokaryotic vector for expression of dsRNA

Before construction of the expression vector, the functional domain of the *CiHR3* gene was predicted by using NCBI protein BLAST, especially ligand-binding domain (LBD), which is necessary to maintain insect growth and development (Montagne et al. 2010). For the *CiHR3* target gene, two interference fragments were constructed *viz*., fragment one (F1 and R1), and fragment two (F2 and R2). Enhanced Green Fluorescent Protein (EGFP) gene served as a negative control (F3 and R3). The vector L4440 was used with host strain *E. coli HT115*. All PCR products of interference fragments and L4440 vector were digested with *Xho*I and *Kpn*I and ligated using T4 DNA ligase at 16 ^°^C overnight. The positive clones were then selected and sequenced.

### Synthesis of dsRNA in bacteria

Total RNA was isolated from the last instar stage of *C. infuscatellus* using Trizol reagent (Invitrogen Life Technologies, www.invitrogen.com). Then, two target fragments of 300–600 bp were amplified using gene-specific primers (Table 1) designed on the basis of *CiHR3* gene sequences of *C. infuscatellus*. Similarly, primers of *EGFP* gene (negative control) were also designed (Table 1). The PCR products were first ligated to pMD19-T simple vector and then digested with *Xho*I and *Kpn*I in order to construct recombinant vectors L4440-CiHR3-I1, L4440-CiHR3-I2, and L4440-EGFP. An empty L4440 vector consisted of two convergent T7 polymerase promoters in opposite orientations. The L4440 vector containing PCR products was transformed into competent HT115 (DE3) cells, an RNase III deficient *E. coli* strain with IPTG-inducible T7 polymerase activity. The single colonies of *E. coli* containing L4440 vector with insert were inoculated into LB medium containing ampicillin and cultured overnight. The bacterial solution was diluted to 100× with LB medium and grown to OD_600_ = 0.6. IPTG was added to a final concentration of 0.8 mM, and the culture was incubated with shaking for eight hours at 37 ^°^C. The solution was then sonicated on ice for 20 min and stored at -20 ^°^C until use.

**Table 1.**
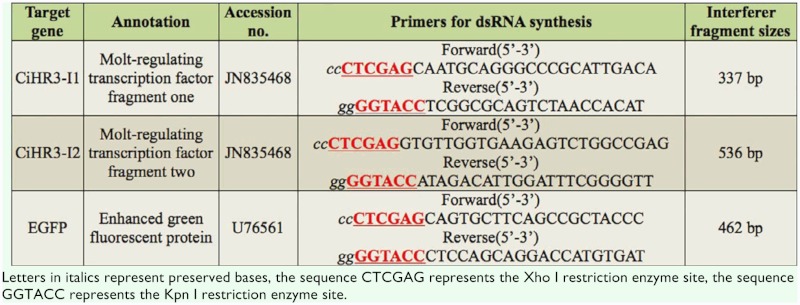
Annotation, GenBank database accession numbers, primers for synthesis of dsRNA, and sizes of interference fragments tested in this study.

### Identification of dsRNA produced in *E. coli* HT115

To analyze the dsRNA synthesized in the bacteria, HT115 (DE3), total RNA was extracted from the bacterial cells using TRI reagent (Sangon Biotech, www.sangon.com). The RNA was treated with DNase I (Takara, www.takarabio.com) to remove the DNA contamination. The nucleic acid pellets were re-suspended in double distilled water and loaded onto a 1.5% agarose/TBE gel. The gel was stained with nucleic acid stain (Goldview®, SBS Genetech Co., Ltd., www.sbsbio.com) and photographed. Sample volumes were normalized according to the concentration of the RNA, and about 4 µg of total RNA was loaded into each lane of the gel.

### Synthesis of dsRNA *in vitro*

The dsRNAs were also synthesized *in vitro* using the ScriptMAX™ Thermo T7 Transcription Kit (TOYOBO, www.toyoboglobal.com). The recombinant plasmids L4440-CiHR3-I1, L4440-CiHR3-I2, and L4440-EGFP were extracted from the HT115 (DE3) cells with AxyPrep™ Plasmid Miniprep Kit (Axygen, www.axygen.com). A high-fidelity DNA polymerase (Takara, www.takarabio.com) was used to conduct PCR using the plasmid DNA and T7 (TAA TAC GAC TCA CTA TAG GG) primer. PCR conditions were 94 ^°^C for 3 min followed by 35 cycles of 94 ^°^C for 30 sec, 55 ^°^C for 30 sec, and 72 °C for 1 min, and a final extension step at 72 ^°^C for 10 min. PCR products were purified using a PCR purification kit (Tiangen Biotech, www.tiangen.com) and used as a template for synthesis of dsRNA. The quality of the dsRNA was tested by electrophoresis and quantified using a spectrophotometer (NanoDrop Technologies, www.nanodrop.com). The dsRNA was diluted in RNase-free H_2_O to 4–5 µg/µL and stored at -80 ^°^C before use.

### Insect bioassay

The dsRNA-mediated gene silencing was conducted by voluntary feeding bioassays. Third and fifth instar *C. infuscatellus* larvae were starved for 12 hours before the initiation of feeding bioassays. Bioassays were carried out in two groups. One group was treated with bacterial-expressed dsRNA and the other group was treated with *in vitro* synthesized dsRNA. For each group, the bioassays were carried out in four treatments *viz.,* L4440-CiHR3-I1, L4440-CiHR3-I2, L4440-EGFP, and water with three replications. The seven larvae with similar body size and weight were taken in one petri dish for each replication. Either 200 µL of bacterial expressing dsRNA or 50 µg of *in vitro* synthesized dsRNA (diluted in 200 µL double-distilled water for each delivery) was spread on fresh and tender corn kernels (with the similar weight in all treatments) in each petri dish. After treatment, petri dishes were placed in an incubator (25 – 2 ^°^C, 70% RH, 16:8 L:D). All bioassays were carried out for seven days. The insects were treated for six consecutive days. On each day, insects from one petri dish were gently transferred with soft hair brush to a new petri dish containing treated fresh and tender corn kernels. The average body weight of larvae was recorded for 1–7 days in both groups. The *EGFP* dsRNA served as a negative control and water treatment served as a control for bacterial expressed dsRNA experiments. For *in vitro* dsRNA experiments, controls were fed with the *EGFP* dsRNA.

### Quantitative real-timePCR (qRT-PCR) and reference gene selection

qRT-PCR was performed in an iCycler iQ RT-PCR detection system with iQ™ SYBR® Green supermix (Bio-Rad, www.biorad.com). The Trizol reagent (Invitrogen Life Technologies, www.invitrogen.com) was used to isolate total RNA from three individual insects on the 7^th^ day after treatment. The total RNAs were treated with DNase I (Takara, www.takarabio.com). cDNA was synthesized using PrimeScript™ cDNA synthesis kit (Takara, www.takarabio.com). DNase I-treated total RNA was used as a template. Each qRT-PCR reaction mixture (10 µL volume) consisted of 5 µL of FastStart SYBR Green Master (Roche Diagnostics, www.roche.com), 1.2 µL of cDNA, and 0.6 µL each of forward and reverse gene-specific primers. An initial incubation was 95 ^°^C for 3 min, followed by 40 cycles of 95 ^°^C for 10 sec, 55 ^°^C for 20 sec, and 72 ^°^C for 30 sec, and a final extension at 72 ^°^C for 10 min. A fluorescence reading determined the extension of amplification at the end of each cycle. Each experiment was repeated three times using samples from independent treatments. The similar quantitative primers were used for the two interference fragments, *EGFP* as a negative control, and water as a control: CiHR3-qRT-PCR-P1: 5′- AAC CAC CGC CAC AGC AGC CTT AC -3′; CiHR3-qRT-PCR-P2: 5′- GAT GTC TCC CTC CGC GTG ACC AA -3′. To detect mRNA level of *CiHR3* gene, the *β*-actin gene of *C. infuscatellus* was evaluated with Bestkeeper software package. The primers for *β*-actin gene (GenBank no. JN985905) were: Ci-*β*-actin-P1: 5′- ACC AAC TGG GAC GAT ATG GAG AA -3′, Ci-*β*-actin-P2: 5′- CCT CAG TCA AGA GGA CTG GGT GC -3′. The relative expression levels for specific genes as compared to the *β-*actin reference gene were calculated by the 2_ΔΔCT _method_ _as_ described by Livak and Schmittgen (2001).

### Statistical analysis

Statistical analysis was carried out using SPSS software (SPSS v.13.0, www.brothersoft.com). The knockdown efficiency test and the overall effects of RNAi on the growth and development of insects were analyzed using Student's *t-*test. The means were separated by using Duncan multiple mean separation techniques.

## Results

### *CiHR3* gene serves as an interference fragment

**Figure 1.**
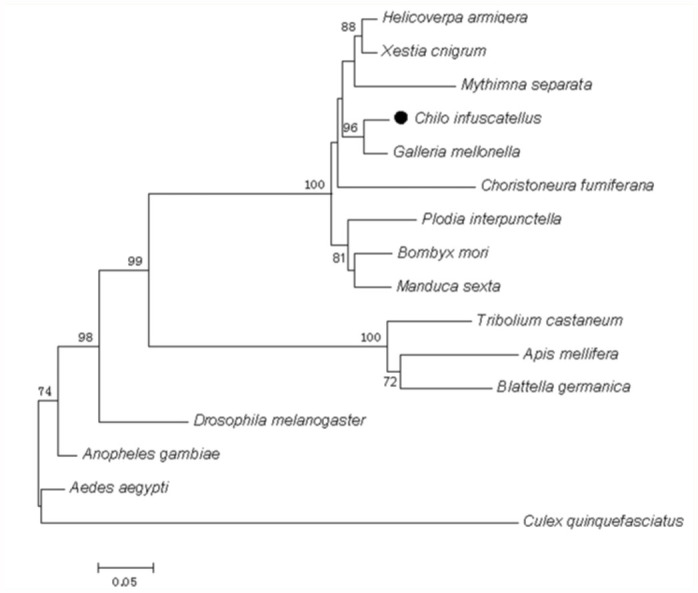
A phylogenetic tree constructed from the amino acid sequences of CiHR3 and 15 related insect molt-regulating transcription factors aligned by ClustalW. A phylogenetic tree was constructed using MEGA4.0.2 software applying the method of Neighbor-Joining (NJ). The data set was subjected to 1000 bootstrap replicates. The bootstrap values higher than 50 are indicated at the corresponding branch. The bar represents 0.05 substitutions per 100 amino acids. Insect species and GenBank accessions are: *Aedes aegypti*, AAF36970; *Galleria mellonella*, AAA83401; *Bombyx mori*, NP_001037012; *Plodia interpunctella*, AAT08962; *Mythimna separata*, ACZ71480; *Xestia cnigrum*, ACZ71479; *Tribolium castaneum*, XP_974561; *Apis mellifera*, XP_392128; *Blattella germanica*, CAJ90623; *Helicoverpa armigera*, ACH86113; *Drosophila melanogaster*, AAA28461; *Choristoneura fumiferana*, AAC47163; *Manduca sexta*, CAA52654; *Culex quinquefasciatus*, XP_001865834; *Anopheles gambiae*, XP_319750; *Chilo infuscatellus*, JN835468. High quality figures are available online.

*CiHR3* gene belongs to the family of molt-regulating transcription factors. The molting process in *C. infuscatellus* is regulated by the hormone-mediated cascade reaction consisting of a series of genes, and ecdysone regulation of transcription factor is a direct product of early induced gene expression (Zhao et al. 2004). The expression of this gene was immediately activated after a specific sequence on its target DNA was bound and directly involved in the late ecdysone-induced gene regulated by a molting cascade in the key factor. A phylogenetic tree constructed from the amino acid sequences of CiHR3 and other 15 related insect molt-regulating transcription factors indicated that CiHR3 is closely related to *HR3* genes from lepidopteron insects, especially *Galleria mellonella* (Figure 1).

### Verification of dsRNA synthesis in bacteria

**Figure 2.**
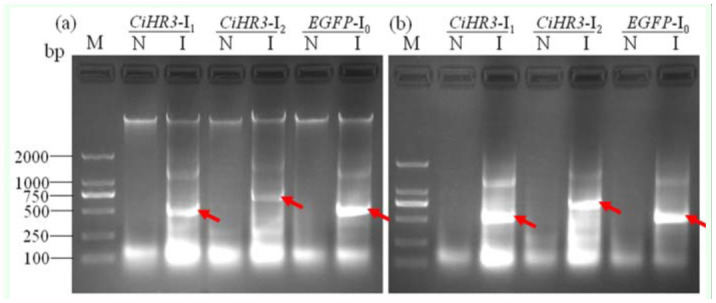
Identification of dsRNA of *CiHR3* and *EGFP* genes produced in bacteria. Total RNA was extracted from bacteria using bacterial RNA special extraction kit, and the RNA was treated with DNase I to remove the contamination of genomic DNA. The nucleic acid pellets were dissolved in RNase free H2O, loaded onto a 1.5% agarose/TBE gel, stained with nucleic acid stain and photographed, (a) RNA treated with DNase I treatment and (b) RNA without DNase I treatment. N, the lane with an RNA sample extracted from bacteria that were not treated with IPTG; I, the lane with an RNA sample extracted from bacteria that were treated with IPTG. The position of dsRNAs produced is marked with arrow heads. High quality figures are available online.

The PCR products of two interference fragments and *EGFP* gene as a negative control were cloned into L4440 vector digested by *Xho*I and *Kpn*I, separately. The resulting plasmids were transformed into HT115 (DE3) competent cells. After adding IPTG into the bacterial growth medium, the dsRNA of the target gene was induced. Thus, dsRNAs of all target gene fragments were successfully synthesized in the bacteria (Figure 2).

### The effects of feeding dsRNAs on larval growth of *C. infuscatellus*

**Figure 3.**
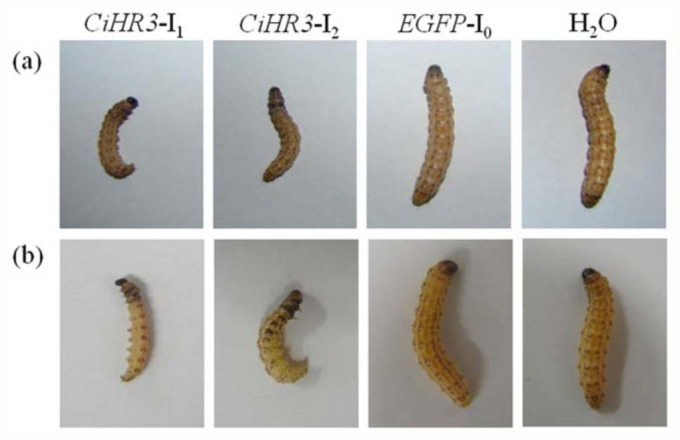
The effects of feeding *CiHR3* dsRNA to the 3^rd^ instar larvae of *Chilo infuscatellus* after feeding the bacterial-expressed dsRNA (a) or *in vitro* synthesized dsRNA (b). High quality figures are available online.

**Figure 4.**
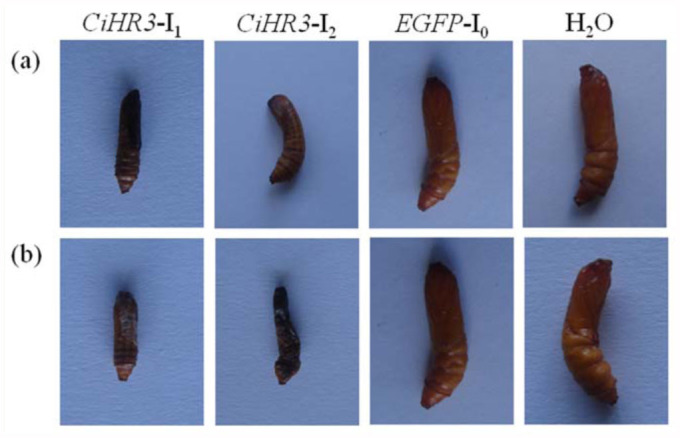
The effects of feeding *CiHR3* dsRNA to the 5^th^ instar larvae of *Chilo infuscatellus* after feeding the bacterial-expressed dsRNA (a) or *in vitro* synthesized dsRNA (b). High quality figures are available online.

**Figure 5.**
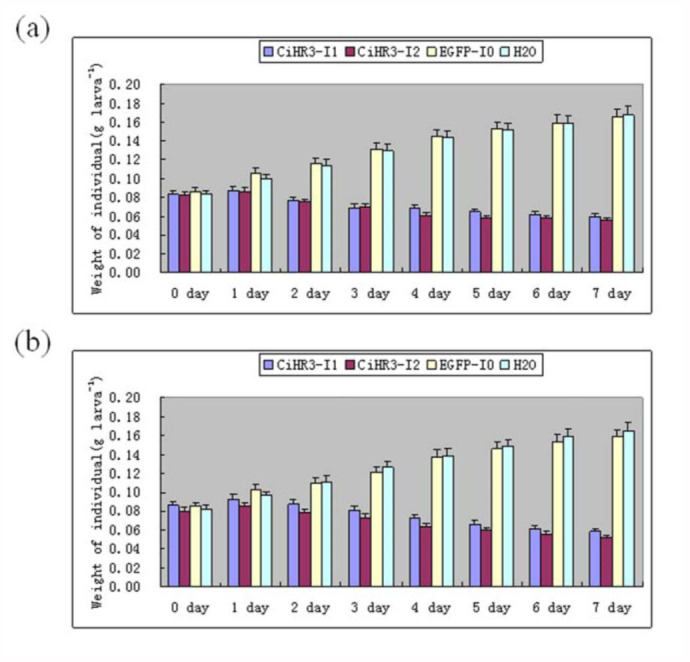
The effects of RNAi on larval growth of *Chilo infuscatellus* after feeding the bacterial-expressed dsRNA(a) or *in vitro* synthesized dsRNA(b). The blue bars represent CiHR3-I1, the red bars represent CiHR3-I2, the yellow bars represent EGFP-I0, and the green bars represent H_2_O: Weights of individual insect in the control and treatment groups within 0, 1, 2, 3, 4, 5, 6, and 7 days were analyzed. High quality figures are available online.

The effects of target genes on *C. infuscatellus* by feeding bacterial-expressed dsRNA were tested. The phenotypic effects were observed as a decrease in larval body weight. The body weight of the 3^rd^ instar larvae of *C. infuscatellus* was significantly decreased when feeding on bacteria-expressed *CiHR3* dsRNA as compared with that of negative control or water treatment, and 25% insects died before entering into the 4^th instar stage (Figure 3). Feeding *CiHR3* dsRNA to the 5th^ instar larvae of *C. infuscatellus* caused larval body weight to significantly decrease and 20% insects failed to enter into the pupal stage (Figure 4). However, there were no significant differences in the decrease in body weight between the treatments with bacterial expressed dsRNA and *in vitro* synthesized *EGFP* dsRNA (negative control) (Figure 5). Two interference fragments of *CiHR3* gene were selected, *viz*., CiHR3-I1 and CiHR3-I2. The CiHR3-I2 fragment represents LBD domain of *CiHR3* gene. The bioassay results indicated that feeding of CiHR3-I2 dsRNA was more effective than that of CiHR3-Il dsRNA, but there was no significant difference between them.

### Knockdown efficiency after feeding bacteria-expressed and *in vitro* synthesized dsRNAs

**Figure 6.**
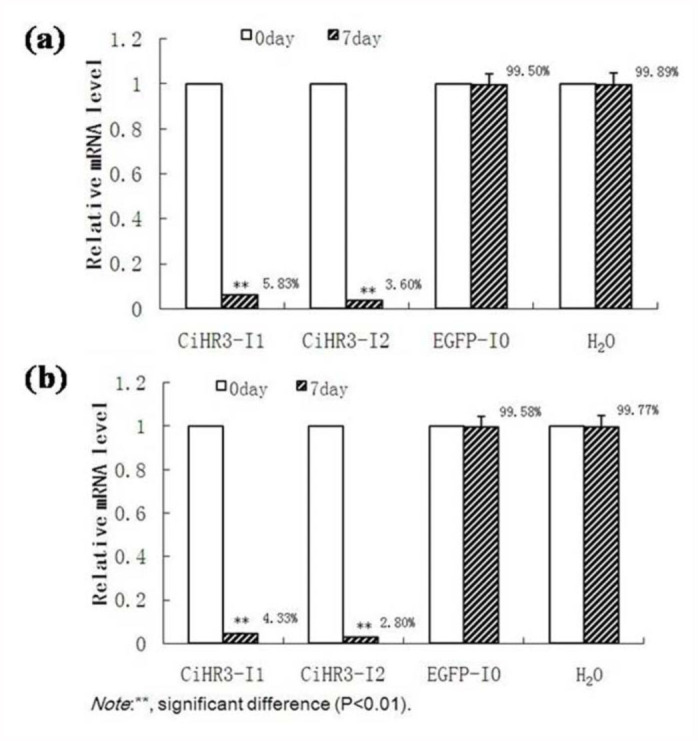
The effect of feeding RNAi on target-gene expression at the 7^th^ day of bacterial-expressed dsRNA (a) or the *in vitro* synthesized dsRNA (b). The results are expressed as the relative expression level of the target gene in treated samples (7^th^ day) compared with that in controls (0 day). The values are shown as mean ± SD (n = 5–8). The values at the top of the bars show the knockdown percentage. The statistical significance of the gene expression between two samples was evaluated using Student's *t*-test. Asterisks represent significant differences between controls and treatments at *p* < 0.05. High quality figures are available online.

To test the molecular effect of feeding RNAi on target-gene expression, qRT-PCR was conducted with individual samples at seven days after feeding with the bacteria-expressed dsRNA (Figure 6a) or *in vitro* synthesized dsRNA (Figure 6b). For bacterial expressed dsRNA experiments, expression of target genes was reported as the proportion of that in control samples fed with bacterial-expressed dsRNA. For *in vitro* dsRNA experiments, expression of target genes was calculated as the ratio of that in controls fed with the dsRNA using reporter *EGFP* gene as a template. All expression levels were normalized to *β*-actin. The common *β*-actin housekeeping gene for qRT-PCR analysis was selected. *β*-actin was chosen as the reference gene to calculate relative expression levels of target genes because it showed the most stable expression among samples. The feeding of bacterially expressed *CiHR3* dsRNA showed a more efficient knockdown than *in vitro* synthesized dsRNA.

## Discussion

The most significant finding of this study is the discovery that feeding of *CiHR3* dsRNA expressed in bacteria could be used to silence the molt-regulating transcription factor gene, a key gene related to growth and development in *C. infuscatellus*. In this study, both the bacterial-expressed and the *in vitro* synthesized dsRNA delivered by feeding successfully triggered the knockdown of *CiHR3* gene and caused significant weight loss in insects as compared to negative control and water treatment as a control, suggesting an effective RNAi response in *C. infuscatellus*.

In insect ecdysteroid signaling cascades, there are two important transcriptional regulators: E75 and HR3. HR3 activates target downstream genes in the signaling pathway (King-Jones and Thummel 2005). In *Drosophila melanogaster*, DHR3 re-sets the genetic cascade triggered by 20E at level of the early genes, repressing their expression at the prepupal stage, while activating the nuclear receptor bFTZ-F1, which provides competence to respond to 20E in late prepupal stage (Lam et al. 1997). Earlier studies demonstrated that DHR3 participates in a hierarchal regulatory circuit in response to ecdysone signaling (Sullivan and Thummel 2003), but also acts in a negative feedback loop to repress ecdysone receptor-mediated signaling (White et al. 1997). Recent studies in *Drosophila* have shown that they have generated LBD-specific DHR3 mutants and demonstrated that the LBD of DHR3 is necessary to maintain insect growth and development (Montagne et al. 2010). The ligand-binding domain (LBD) of HR3, a family of ligand-activated transcription regulators, which regulate various physiological functions in metazoans, from development and reproduction to homeostasis and metabolism (Marchler-Bauer et al. 2011), contains histidine and cysteine residues situated in key positions to bind a heme moiety (Reinking et al. 2005).

In *C. elegans*, the dsRNA-mediated gene silencing works systemically by injection, feeding, or soaking (Timmons et al. 2001). The mechanisms of RNAi signal transport and/or amplification are known to be mediated by two important proteins, RNA-directed RNA polymerase (RdRP) and systemic RNAi defective (SID) (Jose et al. 2007; Xu and Han 2008). However, SID insects apparently lack an RdRp-based mechanism, so SID-based passive transport is likely the mechanism of RNAi signaling in insects. But, it also needs further investigation (Gordon and Waterhouse 2007). Although the RNAi mechanism in insets is not clear, feeding RNAi has already been considered as a potentially important tool for amplifications in pest control (Tian et al. 2009; Runo et al. 2011; Zhu et al. 2011). The data presented here shows that the efficient induction of RNAi using bacteria to deliver dsRNA is a possible method that could be used for the control of *C. infuscatellus*. The most significant advantage of this method is that it is cheaper as compared to *in vitro* synthesis of dsRNA by Kit. Large-scale production of bacterial expressing dsRNA for use as an insecticide to spray on the crop could become a reality in the near future. The use of bacterially produced dsRNA for pest management also needs to overcome some obstacles, such as public acceptance. Another problem is that to release bacterial-expressed dsRNA, *E*.*coli* cell walls need to be destroyed, usually by sonication or heat. In fact, the stability of dsRNA in the open environment should also be considered carefully.

The molt-related transcription factors have been targeted commonly for pest control due to its key role in insect growth and development. The bioassay results indicated that feeding of CiHR3-I2 dsRNA containing LBD domain of *CiHR3* gene was more effective than that of CiHR3-I1 dsRNA, but there was no significant difference between them. Generally, most of the chemical insecticides interfering with the molting process of insects are more lethal if applied at earlier stages. In the present study, we studied the effect of dsRNA only for seven days, so study of the whole life cycle of the insects including reproductive parameters treated at earlier stages is needed. The findings in the present study would serve as foundation to explore alternative strategies to control *C. infuscatellus*.
